# UDSMProt: universal deep sequence models for protein classification

**DOI:** 10.1093/bioinformatics/btaa003

**Published:** 2020-01-08

**Authors:** Nils Strodthoff, Patrick Wagner, Markus Wenzel, Wojciech Samek

**Affiliations:** Department of Video Coding & Analytics, Fraunhofer Heinrich Hertz Institute, Berlin 10587, Germany

## Abstract

**Motivation:**

Inferring the properties of a protein from its amino acid sequence is one of the key problems in bioinformatics. Most state-of-the-art approaches for protein classification are tailored to single classification tasks and rely on handcrafted features, such as position-specific-scoring matrices from expensive database searches. We argue that this level of performance can be reached or even be surpassed by learning a task-agnostic representation once, using self-supervised language modeling, and transferring it to specific tasks by a simple fine-tuning step.

**Results:**

We put forward a universal deep sequence model that is pre-trained on unlabeled protein sequences from Swiss-Prot and fine-tuned on protein classification tasks. We apply it to three prototypical tasks, namely enzyme class prediction, gene ontology prediction and remote homology and fold detection. The proposed method performs on par with state-of-the-art algorithms that were tailored to these specific tasks or, for two out of three tasks, even outperforms them. These results stress the possibility of inferring protein properties from the sequence alone and, on more general grounds, the prospects of modern natural language processing methods in omics. Moreover, we illustrate the prospects for explainable machine learning methods in this field by selected case studies.

**Availability and implementation:**

Source code is available under https://github.com/nstrodt/UDSMProt.

**Supplementary information:**

Supplementary data are available at *Bioinformatics* online.

## 1 Introduction

Inferring protein properties from the underlying sequence of amino acids (primary structure) is a long-standing theme in bioinformatics and is of particular importance in the light of advances in sequencing technology and the vast number of proteins with mostly unknown properties. A rough estimate for this number is given by the size of the sparsely annotated TrEMBL dataset (158M) and should be set into perspective by comparison to the size of well-curated Swiss-Prot (The UniProt Consortium, 2018) dataset (560K) with a much more complete annotation of the protein properties.

There is a large body of literature on methods to infer protein properties, most of which make use of additional handcrafted features in addition to the primary sequence alone (Cozzetto *et al.*, 2016; Dalkiran *et al.*, 2018; Gong *et al.*, 2016; Håndstad *et al.*, 2007; Li *et al.*, 2017, 2018; Shen and Chou, 2007). These features include experimentally determined functional annotations (such as Pfam; El-Gebali *et al.*, 2019) and information from homologous, i.e. evolutionary-related proteins. The latter are typically inferred from well-motivated but still heuristic methods such as the basic local alignment search tool (BLAST; Madden, 2013) that searches a database for proteins that are homologous to a given query protein, via multiple sequence alignment. Handcrafted features based on experimental results rely on a preferably complete functional annotation and are therefore likely to fail to generalize for incompletely annotated proteins (Price *et al.*, 2018). Handcrafted features derived from multiple sequence alignments rely on alignment algorithms that typically scale at least linearly with query and database size. This time complexity is not able to keep up with the present size and the exponential growth rates of present protein databases.

These bottlenecks urge for the development of methods that allow to directly predict protein properties from the sequence of amino acids alone, which is, therefore, a topic on the agenda of many research institutions (Bileschi *et al.*, 2019; Rao *et al.*, 2019; Rives *et al.*, 2019). Methods from deep learning, and, in particular, self-supervised algorithms from natural language processing (NLP), are promising approaches in this direction.

The machine learning (ML) community recently gained interest in protein classification as possible application area for deep learning methods (see e.g. AlQuraishi, 2019; Bileschi *et al.*, 2019; Rao *et al.*, 2019; Rives *et al.*, 2019; Upmeier zu Belzen *et al.*, 2019). In NLP, self-supervised approaches have shown tremendous prospects across a wide variety of tasks (Devlin *et al.*, 2019; Howard and Ruder, 2018; Liu *et al.*, 2019; Peters *et al.*, 2018; Radford *et al.*, 2018, 2019; Song *et al.*, 2019; Yang *et al.*, 2019), which rely on leveraging implicit knowledge from large unlabeled corpora by pre-training using autoregressive language modeling or autoencoding tasks. This approach goes significantly beyond the use of pre-trained word embeddings, where only the embedding layer is pre-trained, whereas the rest of the model is initialized randomly.

Protein classification tasks represent a tempting application domain for such techniques exploiting the analogy of amino acids as words, protein domains as sentences and proteins as text paragraphs. In this setting, global protein classification tasks, such as enzyme class prediction, are analogous to text classification tasks (e.g. sentiment analysis). Protein annotation tasks, such as secondary structure or phosphorylation site prediction, map to text annotation tasks, such as part-of-speech tagging or named entity recognition. Although this general analogy has been recognized and exploited already earlier by Asgari and Mofrad (2015), self-supervised pre-training is a rather new technique in this field. Existing literature approaches in this direction (Rao *et al.*, 2019; Rives *et al.*, 2019) show significant improvements for models that were pre-trained using self-supervision compared with their counterparts trained from scratch on a variety of tasks and demonstrate that models leverage biologically sensible information from pre-training. However, none of them explicitly demonstrated that pre-training can bridge the gap to state-of-the-art approaches that mostly rely on handcrafted features such as position-specific scoring matrices (PSSMs) derived via BLAST.

Our main contributions in this article are the following: (i) we put forward a universal deep sequence model for protein classification (*UDSMProt*) that is pre-trained on Swiss-Prot and fine-tuned on specific classification tasks without any further task-specific modifications. (ii) We demonstrate that this model is able to reach or even surpass the performance level of state-of-the-art classification algorithms many of which make use of PSSM features. This indicates the feasibility of inferring protein properties from the sequence alone across a variety of different tasks. (iii) We demonstrate the particular effectiveness of our approach for small datasets.

## 2 Algorithms and training procedures

### 2.1 UDSMProt: universal deep sequence models for protein classification

The idea of *UDSMProt* is to apply self-supervised pre-training to a state-of-the-art recurrent neural network (RNN) architecture using a language modeling task. In this way, the model learns implicit representations from unlabeled data that can be leveraged for downstream classification tasks. We aim to address a range of different classification problems within a single architecture that is universal in the sense that only the dimensionality of the output layer has to be adapted to the specific task. This facilitates the adaptation to classification tasks beyond the three exemplary tasks considered in this work. For fine-tuning on the downstream classification tasks, all embedding weights and long short-term memory (LSTM) weights are initialized using the same set of weights obtained from language model pre-training. As we will demonstrate, this is a particularly powerful choice for small datasets.

Presently, there are two main objectives for self-supervised pre-training in NLP, autoregressive language modeling and autoencoding (see Yang *et al.*, 2019 for a concise introduction to both concepts). Autoregressive language modeling is an inherently unidirectional approach whereas autoencoding directly incorporated bidirectional context. One notable example of the autoregressive category is the RNN-based *AWD-LSTM* language model (Merity *et al.*, 2018) compared with *BERT* (Devlin *et al.*, 2019) as main proponent for the autoencoding category. Autoregressive approaches, such as *ULMFit* (Howard and Ruder, 2018), can be trained with considerably smaller computational budget than autoencoding, transformer-based architectures, while still showing very competitive performance on NLP text classification tasks (see e.g. Xie *et al.*, 2019) and are, therefore, the method of choice for our intended task.

**Fig. 1.**
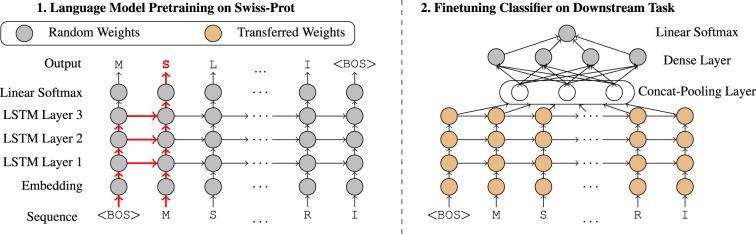
Schematic illustration of the training procedure, here for the amino acid sequence MSLR…RI. The <BOS>-token marks the beginning of the sequence. The red arrows show the context for forward language model for predicting next character (S) given sequence <BOS>M of length 2. For fine-tuning on the downstream classification tasks, all embeddings weights and LSTM weights are initialized using the same set of weights obtained from language model pre-training. This has to be contrasted with the use of pre-trained embeddings, where just the embedding weights are initialized in a structured way before the downstream fine-tuning step

Our proposed method relies on an *AWD-LSTM* language model (Merity *et al.*, 2018), which is, at its heart, a three-layer LSTM regularized by different kinds of dropouts (embedding dropout, input dropout, weight dropout, hidden state dropout and output layer dropout). During language model training, gradients are backpropagated using backpropagation through time (BPTT) with variable length sequences as in (Merity *et al.*, 2018) using batches of ∼70 tokens and the output layer remains tied to the weights of the embedding layer. For classifier training, we use BPTT for text classification (Howard and Ruder, 2018), where gradients are accumulated potentially over multiple batches without resetting the LSTM’s hidden state and backpropagated explicitly up to a maximum context of 1024 tokens. Specific model parameters are listed in Supplementary Table S2. The training procedure for transfer learning is largely inspired by *ULMFit* and proceeds as follows: In a first step, we train a language model on the Swiss-Prot database. In a second step, the language model’s output layer is replaced by a concat-pooling layer (Howard and Ruder, 2018) and two fully connected layers (see Fig. 1 for a schematic illustration). When fine-tuning the classifier, we gradually unfreeze layer group by layer group (four in total) for optimization, where we reduce the learning rate by a factor of two compared with the respective previous layer group (Howard and Ruder, 2018). A single model is by construction only able to capture the context in a unidirectional manner, i.e. processing the input in the forward or backward direction. As simplest approach to incorporate bidirectional context into the final prediction, we train separate forward and backward language models with corresponding fine-tuned classifiers. An ensemble model is obtained by averaging the output probabilities of both classifiers. We use a one-cycle learning rate schedule (Smith, 2018) during training for 30 epochs in the final fine-tuning step. All hyperparameters were optimized based on the model performance on a separate validation set, while we report performance on a separate test set. Our way of addressing the specific challenges of the remote homology datasets are described in Section 3.4. In all cases, we use binary/categorical crossentropy as loss function and the AdamW optimizer (Loshchilov and Hutter, 2019). Note that a potential intermediate step where one fine-tunes the generic language model on the corpus underlying the classification step, as proposed by Howard and Ruder (2018), did only show an improvement in terms of language model quality but did not result in an improved downstream classification performance. This step was therefore omitted for the results presented below.

### 2.2 Baseline model

In our experiments below, we mostly compare directly to reported results from approaches in the literature on predefined datasets. However, this does not allow for in-depth comparisons that modify for example details of the training procedure. To still allow to relate the results of the proposed method to state-of-the-art performance, we use a baseline model that reaches state-of-the-art performance on literature benchmarks and that can henceforth be used as proxy for models considered in the literature.

The performance of literature approaches on many protein classification tasks has been driven to a large extend by the inclusion of different kinds of handcrafted features rather than sophisticated model architectures or training procedures. The most beneficial input features throughout a variety of different classification tasks are obviously the PSSMs based on a multiple sequence alignment computed via position-specific iterative BLAST (PSI-BLAST; Madden, 2013). PSI-BLAST can compare query sequences with a given sequence database and returns a list of local alignments solved with heuristics instead of optimal local alignments solved with the more time-consuming Smith–Waterman algorithm. PSI-BLAST is then used to find more distant relatives of a query protein, where a list of closely related proteins is created to get an initial general profile sequence. This profile sequence is used as a new query for the next iteration where a larger list of proteins is found for which again a profile sequence is computed. This process is repeated to a desired number of iterations. In our experiments, we used the same parameters as reported in the literature (Dalkiran *et al.*, 2018; Li *et al.*, 2018; Shen and Chou, 2007), namely three iterations with an e_value of 0.001 as threshold for which an alignment is considered as significant. Although the raw sequences from Swiss-Prot contain 20 standard and six non-standard amino acids, PSSM features are computed only for the 20 standard amino acids. The raw sequence of length *L* was then one-hot encoded into an L×26 matrix which is concatenated with the L×20 PSSM feature matrix yielding an L×46 input matrix overall. To make use of the full parallelization capabilities while retaining most information, we padded the sequences to a maximum length of 1024 residues.

For all following experiments, we used a convolutional neural network (CNN) with seven layers. Each convolutional layer was followed by a rectified linear unit and max pooling by a factor of 2. The number of filters across layers is: 1024, 512, 512, 512, 256, 256 and 256 (with valid padding mode) each with a filter size of 3. The convolutional stage was followed by flattening layer and three dense layers (512, 256 and 128) each followed by dropout (with 25% dropout rate) and finally a softmax layer with nodes for each class (e.g. six nodes for Level 1 enzyme prediction). For all models, we minimized categorical crossentropy with AdaMax, a variant of adaptive moment estimation (Adam) based on the infinity norm (Kingma and Ba, 2015), which lead to slightly better results in our CNN experiments compared with the original Adam optimizer. The hyperparameters follow those provided in this article.

## 3 Results and discussion

The results are organized as follows: we discuss language modeling as baseline task in Section 3.1 and then demonstrate the capabilities of *UDSMProt* on three prototypical protein classification tasks, namely enzyme class prediction in Section 3.2, gene ontology (GO) prediction in Section 3.3 and remote homology detection in Section 3.4. For enzyme class prediction, we provide an extensive evaluation highlighting several important aspects.

### 3.1 Language modeling

The language modeling task involves predicting the next token for a given sequences of tokens and is one of the key NLP tasks for demonstrating the general understanding of a language. In this particular case, it builds up an implicit knowledge about the structure of proteins, which can potentially be leveraged for downstream classification tasks.

Our character-based language model operates on protein sequence data tokenized on the level of amino acids (see Fig. 1). Interestingly, the language model performance depends strongly on the way the similarity threshold is incorporated in the train–test split procedure. For this reason, we split the data into train, validation and test set with ratios 90:5:5 and compare two methods: (i) random splits without taking sequence similarity into account and (ii) splits according to UniRef50 cluster assignments. Although the model trained on a random split reaches a perplexity of 6.88 and a corresponding next character prediction accuracy of 0.409, the model trained on a cluster-based split only reaches a perplexity of 11.75 with 0.244 accuracy. However, these differences in language model performance do not lead to measurable differences in the downstream performance (see Supplementary Section S3 for a detailed discussion). In Supplementary Section S3, we also investigate the impact of the model architecture on the language performance within several ablation studies. Language model performance metrics inherently depend on the dataset and the vocabulary size and it is hard to estimate their significance. As simplest baseline, language model prediction accuracies can be put into perspective by comparison to random guessing corresponding to an accuracy of 0.04, which conveys that the language model acquired non-trivial knowledge about the underlying construction principles of proteins. We analyze the learned representations in two ways: first, we visualize the learned amino acid embeddings via t-SNE and find good agreement with their known physio-chemical properties (Taylor, 1986). Second, we analyze the model’s outputs after the contact-pooling-layer which hints at the fact that language model pre-training compared with training from scratch leads to a more efficient encoder representation (see Supplementary Section S4).

### 3.2 Enzyme class prediction

We start our analysis on downstream classification tasks with enzyme classification for the reason that it is a conceptually simple task for which a large number of annotated examples are available. The main experiments in this section are organized in a two-step process: first, we analyze the proposed approach on custom datasets in a well-defined experimental environment comparing to a baseline model operating on PSSM features (Section 3.2.2). As a second step, we directly compare to literature results demonstrating the proposed method indeed reaches or even exceeds state-of-the-art performance for this task (Section 3.2.3).

#### 3.2.1 Task and datasets

Enzyme prediction is a functional prediction task targeted to predict the enzyme commission (EC) number from a hierarchical numerical classification scheme for enzymes based on the chemical reactions they catalyze. In particular, we consider discriminating enzyme versus non-enzyme (Level 0), predicting main enzyme class (Level 1) and enzyme subclass (Level 2). A powerful EC classification algorithm of the pre-deep-learning era was provided by *EzyPred* (Shen and Chou, 2007), which owed its success to the design of a hierarchical approach and to appropriate input features, which are a combination of the functional (BLAST against a Pfam database) and evolutionary information (PSI-BLAST against the Swiss-Prot database). For hierarchical classification (Levels 0–2), a simple k nearest neighbor classifier (KNN) was trained in order to achieve convincing results. *EzyPred* was superseded by *DEEPre* (Li *et al.*, 2018) where deep learning was applied to raw sequence and homology data as input and that was recently extended toward multi-functional enzyme classification (Zou *et al.*, 2019). Instead of training simple classifiers on highly engineered features, they trained feature representation and classification in an end-to-end fashion with a hybrid CNN-LSTM-approach. Recently, *ECPred* (Dalkiran *et al.*, 2018) also showed competitive results by building an ensemble of well-performing classifiers (Subsequence Profile Map with PSSM (Sarac *et al.*, 2008), BLAST-KNN (Madden, 2013) and Pepstats-SVM using peptides statistics (Rice *et al.*, 2000). Nevertheless, the drawbacks described in Section 1 remain, i.e. requiring functional annotations of homologous proteins, which are not guaranteed for evolutionary distant or insufficient annotated proteins.

In addition to the existing DEEPre (similarity threshold 40%) and ECPred (similarity threshold 50%) datasets (Dalkiran *et al.*, 2018; Li *et al.*, 2018), we also work with two custom EC40 and EC50 datasets, which provide all cluster members as opposed to only cluster representatives (with similarity threshold 40 and 50%) by combining best practices from the literature for the dataset construction (see Supplementary Section S1 for a detailed description).

#### 3.2.2 Effect of similarity threshold and redundant sequences

In order to investigate the benefits of the proposed approach in comparison to algorithms relying on alignment features, we based our initial analysis on the custom EC40 and EC50 datasets. This approach represents a very controlled experimental setup, where one can investigate the effect of the chosen similarity threshold, the impact of redundant sequences during training and potential sources of data leakage during pre-training in a reliable way.

We base our detailed analysis of the proposed method *UDSMProt* (compared with a baseline algorithm operating on PSSM features) on EC prediction tasks at Level 0 (enzyme versus non-enzyme), Level 1 (main enzyme class) and Level 2 (enzyme subclass). It is a well-known effect that the difficulty of the classification problem scales inversely with the similarity threshold, as a higher similarity threshold leads to sequences in the test set that are potentially more similar to those seen during training. In the extreme case of a random split, i.e. by disregarding cluster information, the test set performance merely reflects the algorithm’s capability to approximate the training set rather than the generalization performance when applied to unseen data. The failure to correctly incorporate the similarity threshold is one of the major pitfalls for newcomers in the field. Here, we perform Levels 0, 1 and 2 prediction on two different datasets, namely EC40 (40%) and EC50 (50% similarity cutoff). Both datasets only differ in the similarity thresholds and the version of the underlying Swiss-Prot databases.

If not noted otherwise, CNN models are trained on representative sequences as this considerably reduces the computational burden for determining PSSM features and is in line with the literature (see e.g. Dalkiran *et al.*, 2018; Li *et al.*, 2018). In contrast, *UDSMProt* is conventionally trained using the full training set including redundant sequences, whereas the corresponding test and validation sets always contain only non-redundant sequences. For the EC50 dataset, non-redundant sequences enlarge the size of the training set from 45 to 114k and from 86 to 170k sequences for Levels 1/2 and 0, respectively. For EC40, the size is enlarged from 20 to 100k and from 46 to 150k for Level 1/2 and 0, respectively.

In Table 1, we compare the two classification algorithms *UDSMProt* and the baseline CNN that were introduced in Section 2 in terms of classification accuracy, which is the default metric considered in the literature for this task. There is a noticeable gap in performance across all experiments between CNN(seq; non-red.) and CNN(seq + PSSM; non-red.) which is a strong indication for the power of PSSM features. This gap can be reduced by the use of redundant sequences from training clusters (CNN(seq)) but still remains sizable. Most importantly, the gap can be closed by the use of language model pre-training. Disregarding the case of the EC40 dataset at Level 0, the best-performing *UDSMProt* outperforms the baseline algorithms that make use of PSSM features. Combining information from both forward and backward context consistently improves over models with unidirectional context. As another observation, pre-training leads to a consistent advantage compared with models trained from scratch that cannot be compensated by increasing the number of training epochs for the models trained from scratch.

**Table 1.** EC classification accuracy on the custom EC40 and EC50 datasets

	Level	EC40			EC50		
		0	1	2	0	1	2
Baseline	Seq; non-red.	0.83	0.38	0.25	0.88	0.71	0.70
	Seq	0.84	0.61	0.47	0.92	0.80	0.79
	Seq+PSSM; non-red.; clean	0.91	0.84	0.72	0.95	0.94	0.91
	Seq+PSSM; non-red.; leak.	**0.92**	0.85	0.71	0.95	0.95	0.92
*UDSMProt*	Fwd; pretr.; non-red.	0.82	0.79	0.71	0.93	0.94	0.92
	Fwd; from scratch	0.87	0.79	0.74	0.94	0.94	0.92
	Fwd; pretr.	0.89	0.84	0.83	0.95	0.96	0.94
	Bwd; pretr.	0.90	0.85	0.81	0.95	0.96	0.94
	Fwd+bwd; pretr.	0.91	**0.87**	**0.84**	**0.96**	**0.97**	**0.95**

*Note*: The best-performing classifiers are marked in bold face.

Fwd/bwd, training in forward/backward direction; seq, raw sequence as input; non-red, training on non-redundant sequences, i.e. representatives only; pretr., using language model pre-training; leak., leakage PSSM features computed on the full dataset.

Table 1 illustrates that the *UDSMProt* classification models benefit from redundant training sequences for the downstream classification task, where the benefit is greater as the similarity threshold decreases. Comparing corresponding results from different similarity thresholds, i.e. results from EC40 to those from EC50, reveals the expected pattern, in the sense that lowering the similarity threshold complicates the classification task as test sequences show smaller overlap with sequences from the training set.

Finally, we wanted to use this task to raise the awareness for the issue of data leakage that has—to the best of our knowledge—not received much attention in the literature. Our point of concern is the common practice of pre-computing features such as PSSMs (or pre-training) on the full dataset disregarding the train–test splits for the downstream classification tasks, which inevitably leads to a systematic over-estimation of the model’s generalization performance by implicitly leveraging information about the test set during the training phase. In an attempt to quantify the size of this effect, we compute two sets of PSSM features, one set computed based on the whole Swiss-Prot database [corresponding classification model: CNN(seq+PSSM; non-red.; leakage)] and a separate set based only on cluster members from the training data [corresponding classification model: CNN(seq+PSSM; non-red.; clean)]. It turns out that the model with PSSM features computed on a consistent train–test split always performs slightly worse than its counterpart that relies on PSSM features computed on the whole dataset. However, from a practical perspective, the effect of test data leakage remains small (see Supplementary Section S3 for a corresponding discussion in the context of LM pre-training). In Supplementary Section S6, we provide a more extensive evaluation of the effect by varying the size of the training database that is used for calculating PSSM features.

To reiterate the main findings of the experiments carried out in this section, the most crucial observation is that language model pre-training is capable of closing the gap in performance between models operating on PSSM features compared with models operating on the sequences alone. The second main observation is that redundant sequences rather than cluster representatives only have a positive impact on the downstream classification training. The most obvious explanations for this observation are inhomogeneous clusters that contain samples with different labels that carry more fine-grained information than a single label per cluster representative.

Finally, data leakage arising from inconsistent train–test splits between pre-training and classification is a possible source of systematic over-estimation of the model’s generalization performance and arises e.g. by pre-computing features (such as PSSM or Pfam features) on the full Swiss-Prot database without excluding downstream test clusters. From our experiments on PSSM features in the context of EC prediction, its effect was found to be small in particular for large pre-training datasets such as Swiss-Prot, but it should be kept in mind for future investigations.

#### 3.2.3 Comparison to literature benchmarks

In order to relate our proposed approach to state-of-the-art methods in literature, we conducted an experiment on two datasets provided by *ECPred* (Dalkiran *et al.*, 2018) and *DEEPre* (Li *et al.*, 2018). One of the purposes of this analysis is to justify our choice of the CNN baseline algorithm by demonstrating that it performs on par with state-of-the-art algorithms that do not make use of additional side-information, e.g. in the form of Pfam features. When comparing to literature results on the *DEEPre* dataset, we exclude models relying on Pfam features from our comparison. Leaving aside the very unfavorable scaling with the dataset size (Bileschi *et al.*, 2019) and possible issues with data leakage due to features computed on the full dataset, methods relying on these features will fail when applied to proteins without functional annotations (see also the discussion in Dalkiran *et al.*, 2018). In fact, a recent study estimated that at least one-third of microbial proteins cannot be annotated through alignments on given sequences (Price *et al.*, 2018). Most notably, this excludes the most elaborate *DEEPre* (Li *et al.*, 2018) model (with 0.96 Level 0, 0.95 Level 1 and 0.94 Level 2 accuracy on the *DEEPre* dataset) and *EzyPred* (Shen and Chou, 2007; with 0.91 Level 0 and 0.90 Level 1 accuracy) from the comparison.

Table 2 shows the results of this experiment (see Supplementary Section S5 for details on the evaluation procedure). Note, that a convolutional model (as our baseline) seemed sufficient when compared with the hybrid model of *DEEPre* [using convolutional layers followed by a recurrent layer (LSTM)] as can been seen in Table 2 where our baseline even surpassed the reported performances (91 versus 88% for Level 0 and 84 versus 82% for Level 1). Also for testing on *ECPred*, our baseline approach yielded competitive results indicating a well-chosen baseline model. These results justify a posteriori our design choices for the CNN baseline model.

**Table 2.** EC classification accuracy on the published *DEEPre* and *ECPred* datasets compared with literature results from *DEEPre* (Li *et al.*, 2018) and *ECPred* (Dalkiran *et al.*, 2018) disregarding models relying on Pfam features

	Level	DEEPre (acc.)			ECPred (mean F_1_)	
		0	1	2	0	1
	*ECPred*	—	—	—	0.96	**0.96**
	*DEEPre* (seq+PSSM)	0.88	0.82	0.43	—	—
	Baseline^a^ (seq+PSSM)	**0.91**	**0.84**	0.59	0.97	0.94
*UDSMProt* ^a^	Fwd; pretr.	0.86	0.81	0.75	0.95	0.93
	Bwd; pretr.	0.86	0.83	0.73	0.97	0.93
	Fwd+bwd; pretr.	0.87	**0.84**	**0.78**	0.97	0.94
	Fwd; pretr.; red.	—	—	—	0.97	0.95
	Bwd; pretr.; red.	—	—	—	0.97	0.95
	Fwd+bwd; pretr.; red.	—	—	—	**0.98**	0.95

*Note*: Results on the *DEEPre* dataset were evaluated using 5-fold cross-validation.

Fwd/bwd, training in forward/backward direction; seq, raw sequence as input; pretr., using language model pre-training.

a Results established in this work.

Turning to the performance of the proposed *UDSMProt*, we find a solid prediction performance reaching state-of-the-art performance reported in the literature for algorithms operating on PSSM features. Considering the results of the previous section, the results on the *DEEPre* dataset represent only the lower bound for the achievable performance as it profits considerably from redundant training sequences, which could, however, not be reconstructed from the given representatives without the underlying cluster assignments. Considering the sizable performance gaps between training on redundant and non-redundant datasets in Table 1, it is even more remarkable that *UDSMProt* already reaches state-of-the-art performance when trained on non-redundant sequences. A notable observation is that our approach outperforms *DEEPre* by a large margin for Level 2 when excluding Pfam features. For *ECPred* we report both the performance for training on the original training set as well as the performance on a redundant training set comprising all corresponding Uniref50 cluster members as shown in the three bottom rows in Table 2. In terms of Level 0 performance, the proposed approach outperforms *ECPred* and it shows competitive performance at Level 1.

To summarize, our baseline model reaches state-of-the-art performance compared with literature approaches disregarding those that incorporate features from functional annotations (such as Pfam) and can therefore be used as proxy for state-of-the-art algorithms in the following investigations. This finding enhances a posteriori also the significance of the results established for the EC40 and EC50 datasets in Section 3.2.2. The proposed *UDSMProt* model is very competitive on both literature datasets.

#### 3.2.4 Impact of dataset size

In this section, we aim to demonstrate the particular advantages of the proposed *UDSMProt*-approach in the regime of small dataset sizes. To investigate this effect in a clean experimental setup, we conducted an experiment with consecutively decreasing training set sizes, while keeping test and validation sets fixed. The hyperparameters were kept fixed to those of the run with full training data.

For this experiment, we used the EC50 dataset as described in Supplementary Section S1 with numbers per class as shown in Supplementary Table S1 and trained a Level 1 classifier for each split. We compared our proposed approach (*UDSMProt* with pre-training and trained from scratch) with baseline models (CNN with PSSM features and CNN on redundant sequences only) for seven different training set sizes measured in terms of clusters compared with the number of clusters in the original training set.

**Fig. 2.**
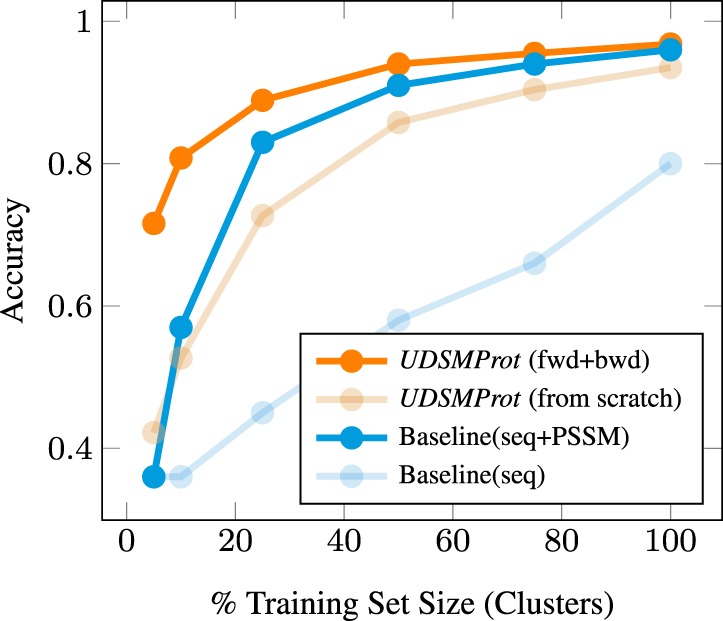
Dependence of the EC classification accuracy (Level 1; EC50 dataset) on the size of the training dataset. *UDSMProt* performs better than the baseline model also in the regime of small datasets that is particularly important for practical applications

The results from Figure 2 show an interesting pattern: the bidirectional *UDSMProt* model always outperforms the CNN baseline model and, most interestingly, the gap between the two models increases for small dataset sizes, which suggests the representations learned during language model fine-tuning represent a more effective baseline for fine-tuning than using PSSMs as fixed input features. As a second observation, also the gap to the models trained from scratch widens. Reducing the number of training clusters by 50% only leads to a decrease in model performance by 3%, whereas the performance of the model trained from scratch drops by 8%.

To summarize, both observations represent strong arguments for applying *UDSMProt* in particular to small datasets. Our results suggest to make language model pre-training a standard procedure in these cases.

### 3.3 GO prediction

To stress the universality of the approach, we now present results for GO prediction, a functional multi-label classification task.

#### 3.3.1 Task and dataset

A more general although closely related problem to enzyme prediction is GO prediction. GO is an international bioinformatics initiative to unify a part of the vocabulary for the representation of proteins attributes. It covers three domains, namely cellular components (CCOs), molecular functions (MFOs) and biological processes (BPOs). The nomenclature is organized into hierarchies ranging from coarse to fine-grained attributes. Similar to enzyme class prediction, the first proposed approaches in this field relied on handcrafted features like functionally discriminating residues with PSSM (Gong *et al.*, 2016) and classification models consisting of an array of support vector machines (Cozzetto *et al.*, 2016). State-of-the-art methods based on neural networks are *DeepGO* (Kulmanov *et al.*, 2018) and *DeepGOPlus* (Kulmanov and Hoehndorf, 2019), where the latter also leverages *Diamond* (BLAST; Buchfink *et al.*, 2015) in an ensemble model. The best-performing method from the CAFA3 challenge (Zhou *et al.*, 2019), *GoLabeler* (You *et al.*, 2018b), is an ensemble method that was recently outperformed by *DeepText2GO* (You *et al.*, 2018a), an ensemble method based on knowledge extraction.

To allow for a direct comparison with the most recent state-of-the-art methods, we work with a dataset constructed using a time-based split (Kulmanov and Hoehndorf, 2019; You *et al.*, 2018b), where the Swiss-Prot annotations before January 2016 are used as training set and those between January 2016 and October 2016 serve as test set (The underlying raw data are available from the data repository accompanying; Kulmanov and Hoehndorf, 2019.). The models are evaluated in each of the three categories MFO, BPO and CCO using the two main metrics of the CAFA3 challenge (Zhou *et al.*, 2019), namely a protein-centric maximum *F*-measure, $F_{\text{max}}$ and $S_{\text{min}}$, which quantifies the semantic distance between predicted and ground truth annotations (see Clark and Radivojac, 2013, for details). These metrics are complemented by the area under the precision-recall-curve that was also reported by You *et al.* (2018a,b) and Kulmanov and Hoehndorf (2019).

#### 3.3.2 Experimental setup and results

As the distribution of GO-labels is very long-tailed, we follow the approach by Kulmanov *et al.* (2018) and Kulmanov and Hoehndorf (2019) and predict only GO-terms that occurred at least 50 times in the training dataset resulting in 5101 unique GO-labels across all three GO categories. As only modification compared with the EC prediction task, we enlarge the size of the hidden layer of the classifier to 1024. We train a single model for all three GO categories optimizing a binary crossentropy loss in this case, since we are dealing with a multi-label classification task. Similarly to (Kulmanov and Hoehndorf, 2019), due to the large size of output layer, we do not explicitly take into account the ontological nature of GO but use a flat output layer of dimension 5101. To illustrate the prospects of ensembling different classifiers, we also report results for ensembling our pre-trained (forward and backward) model with BLAST results from *DiamondScore* using the same relative weighting used by Kulmanov and Hoehndorf (2019).

The results in Table 3 demonstrate the strong performance of *UDSMProt* also in the domain of GO prediction. In particular, the forward–backward model outperforms state-of-the-art methods based on neural networks in terms of $F_{\text{max}}$ for all three GO categories and even reaches a new state-of-the-art result across all considered single-model approaches for $F_{\text{max}}$ in the CCO category as well for all three categories in terms of area under the precision-recall curve (AUPR). Combining its predictions with BLAST-KNN features from *DiamondScore* following Kulmanov and Hoehndorf (2019) leads to very competitive results compared with state-of-the-art ensemble methods in terms of $F_{\text{max}}$ and AUPR even establishing new state-of-the-art results for both metrics in the BPO category. At this point, we would like to stress that the results presented here were obtained without any hyperparameter tuning, using the exact same parameters as for EC prediction apart from changing the dimensionality of the hidden layer, which is in strong contrast to most literature approaches. This suggests that further task-specific hyperparameter tuning might still further enhance the overall performance. The results presented in this section substantiate our claims regarding the universality of transferring implicit knowledge to task-specific requirements.

**Table 3.** GO prediction performance on a dataset based on a time-based split as in (Kulmanov and Hoehndorf, 2019; You *et al.*, 2018b) in comparison to literature results collected by *DeepGOPlus* (Kulmanov and Hoehndorf, 2019)

	Methods	$F_{\text{max}}$			$S_{\text{min}}$			AUPR		
		MFO	BPO	CCO	MFO	BPO	CCO	MFO	BPO	CCO
Single	*Naive*	0.306	0.318	0.605	12.105	38.890	9.646	0.150	0.219	0.512
	*DiamondScore*	0.548	0.439	0.621	8.736	34.060	7.997	0.362	0.240	0.363
	*DeepGO*	0.449	0.398	0.667	10.722	35.085	7.861	0.409	0.328	0.696
	*DeepGOCNN*	0.409	0.383	0.663	11.296	36.451	8.642	0.350	0.316	0.688
Ensemble	*DeepText2GO*	**0.627**	0.441	0.694	5.240	17.713	**4.531**	**0.605**	0.336	**0.729**
	*GOLabeler*	0.580	0.370	0.687	**5.077**	**15.177**	5.518	0.546	0.225	0.700
	*DeepGOPlus*	0.585	0.474	**0.699**	8.824	33.576	7.693	0.536	0.407	0.726
*UDSMProt* ^a^	Fwd; from scratch	0.418	0.303	0.655	14.906	47.208	12.929	0.304	0.284	0.612
	Fwd; pretr.	0.465	0.404	0.683	10.578	36.667	8.210	0.406	0.345	0.695
	Bwd; pretr.	0.465	0.403	0.664	10.802	36.361	8.210	0.414	0.348	0.685
	Fwd+bwd; pretr.	0.481	0.411	0.682	10.505	36.147	8.244	0.472	0.356	0.704
	Bwd+bwd; pretr. + *DiamondScore*	0.582	**0.475**	0.697	8.787	33.615	7.618	0.548	**0.422**	0.728

*Note*: Best overall results (highest $F_{\text{max}}$ and AUPR; lowest $S_{\text{min}}$) are marked in bold face and best single-model results are underlined.

Fwd/bwd, training in forward/backward direction; pretr., using language model pre-training.

a Results established in this work.

On the architectural side, it remains to explore in detail if explicitly incorporating the label hierarchy represents an advantage, which one might suspect from *DeepGO* outperforming its successor *DeepGOCNN*. The rich literature on this subject is reviewed in Silla and Freitas (2010; see also Wehrmann *et al.*, 2018, for a recent deep learning perspective on this subject). Possible solutions range from combining different classifiers via appropriate post-processing procedures, consistency-enforcing loss terms, custom output layers or hierarchical multi-label classification methods (Nakano *et al.*, 2019; Vens *et al.*, 2008). A more detailed analysis is beyond the scope of this article.

### 3.4 Remote homology and fold detection

As third demonstration of the universality of our approach, we consider remote homology detection tasks. The corresponding datasets consist of a few hundred training examples and are thus situated clearly in the small dataset regime investigated in Section 3.2. This substantiates the claims made in Section 3.2 in a real-world setting.

#### 3.4.1 Task and datasets

Remote homology detection is one of the key problems in computational biology and refers to the classification of proteins into structural and functional classes, which is considered to be a key step for further functional and structural classification tasks. Here, we consider remote homology detection in terms of the SCOP database (Murzin *et al.*, 1995), where all proteins are organized in four levels: class, fold, superfamily and family. Proteins in the same superfamily are homologous and proteins in the same superfamily but in different families are considered to be remotely homologous. Remote homology detection has a rich history and the interested reader is referred to a review article on this topic by Chen *et al.* (2018). We will compare to *ProDec-BLSTM* (Li *et al.*, 2017) with a bidirectional RNN operating on PSSM input features building on earlier work (Hochreiter *et al.*, 2007). A classical baseline method is provided by *GPkernel* (Håndstad *et al.*, 2007), who apply kernel-methods to sequence motifs.

For remote homology detection, we make use of the SCOP 1.67 dataset as prepared by Hochreiter *et al.* (2007), which has become a standard benchmark dataset in the field. Here, the problem is framed as a binary classification problem where one has to decide if a given protein is contained in the same superfamily or fold as a given reference protein. The superfamily/fold benchmark is composed of 102/85 separate datasets and we report the mean performance of all models across the whole set. The standard metrics considered in this context are AUC and AUC50, where the latter corresponds to the (normalized) partial area under the ROC curve integrated up to the first 50 false positives, which allows for a better characterization of the classifier in the domain of small false positive rates, which is most relevant for practical applications, than the overall discriminative power of the classifier as quantified by AUC.

#### 3.4.2 Experimental setup and results

The remote homology and fold detection tasks are challenging for two reasons. The datasets are rather small and the task comprises 102 or respectively 85 different datasets that would in principle require a separate set of hyperparameters. To keep the process as simple as possible, we decided to keep a global set of hyperparameters for all datasets of a given task. The procedure is as follows as no validation is provided for the original datasets, we split the training data into a training and a validation set based on CD-HIT clusters (threshold 0.5). We optimize hyperparameters using the mean AUC for all datasets of a given task measured on the validation set. Most importantly, this involves fixing a (in this case constant) learning rate that is appropriate across all datasets. Using these hyperparameter settings, we perform model selection based on the validation set AUC, i.e. for each individual dataset, we select the model at the epoch with the highest validation AUC. We evaluate the test set AUC for these models and report the mean test set metrics.

The results of these experiments are shown in Table 4. Both for homology and fold detection according to most metrics, the *UDSMProt* model trained from scratch performs worse than the original LSTM model (Hochreiter *et al.*, 2007). This is most likely due to the fact that the *UDSMProt* model is considerably larger than the latter model and most datasets are fairly small with a few hundreds training examples per dataset. This deficiency is overcome with the use of language model pre-training, where both unidirectional models perform better than the LSTM baseline model. This observation is in line with the experiments in Section 3.2.4 that demonstrates the particular effectiveness of the proposed approach for small datasets. The best-performing model from the literature, *ProDec-BLSTM*, is a bidirectional LSTM operating on sequence as well as PSSM features. Interestingly, reaching its performance in terms of overall AUC required the inclusion of bidirectional context, i.e. the forward–backward ensemble model. The proposed method also clearly outperforms classical methods such as *GPkernel* (Håndstad *et al.*, 2007) both on the fold and the superfamily level. The excellent results on remote homology and fold detection support our claims on the universality of the approach as well as the particular advantages in the regime of small dataset sizes.

**Table 4.** Remote homology and fold detection performance on the SCOP 1.67 benchmark dataset compared with literature results from *GPkernel* (Håndstad *et al.*, 2007), *LSTM_protein* (Hochreiter *et al.*, 2007) and *ProDec-BLSTM* (Li *et al.*, 2017)

	Methods	Superfamily level		Fold level	
		AUC	${\text{AUC}}_{50}$	AUC	${\text{AUC}}_{50}$
*UDSMProt* ^a^	*GPkernel*	0.902	0.591	0.844	0.514
	*LSTM_protein*	0.942	0.773	0.821	0.571
	*ProDec-BLSTM*	0.969	0.849	—	—
	Fwd; from scratch	0.706	0.552	0.734	0.653
	Fwd; pretr.	0.957	0.880	0.834	0.734
	Bwd; pretr.	0.969	0.912	0.839	0.757
	Fwd+bwd; pretr.	**0.972**	**0.914**	**0.862**	**0.776**

Fwd/bwd, training in forward/backward direction; pretr., using language model pre-training. The best-performing classifiers are marked in bold face.

a Results established in this work.

## 4 Case studies with insights from explainable ML

**Fig. 3.**
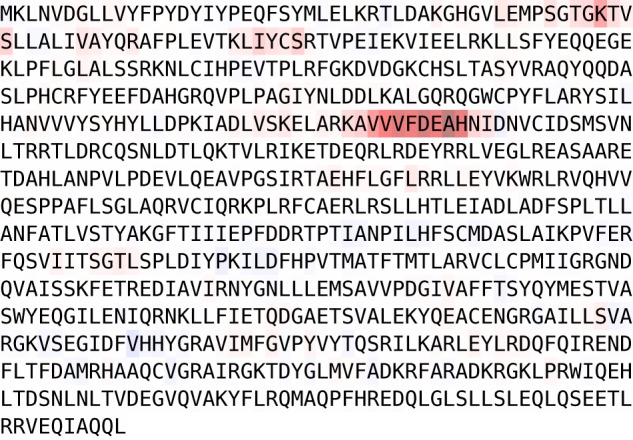
Attribution map for the class EC3 for UniProt accession Q60452 based on integrated gradients. The heatmap shows high relevance on the ‘DEAH’ box (DEAH; Pos. 234–237)

Even though deep neural networks are still often perceived as black-box models, there has been a paradigm shift in the past few years due to the advances in the field of explainable ML. In this section we outline possible applications of interpretability methods to gain deeper insights into the models and the structure of proteins itself (see also Upmeier zu Belzen *et al.*, 2019, for first applications in this direction). Here we focus on *post* *hoc* interpretability methods that return attribution heatmaps in the input space, i.e. the protein sequence itself, that relate to its impact on the classification decision. Attribution methods such as integrated gradients (Sundararajan *et al.*, 2017) relate to parts of the input sequence that influenced the classifier toward/against a certain classification decision. These methods are therefore particularly suited to identify sequence motifs. The representative example in Figure 3 shows this for the case of EC classification, where the classifier identifies a short sequence motif, known as the ‘DEAH’ box, as indicative for EC Class 3. Similarly, the classifier is strongly influenced toward EC Class 6 by regions surrounding the ‘HIGH’ and ‘KMKS’ motifs (see Supplementary Fig. S4). A statistical analysis of these findings is beyond the scope of this manuscript but represents an interesting direction for future research.

Secondly, we revisit the glutaminase example put forward by *DEEPre* (Li *et al.*, 2018). The first and the third isoforms of the amino acid sequence with the UniProt accession number O94925 are enzymes, whereas the second isoform shows no enzymatic activity. This behavior is correctly captured by a model trained on EC Level 1 and no-enzyme data. The attribution map for the first isoform in Supplementary Figure S5 highlights regions beyond position 170 that are not contained in the sequence of the second isoform. The attribution map based on integrated gradients for the second isoform (see Supplementary Fig. S6), already shows high relevance toward the end of the sequence, where the sequence deviates from the canonical sequences. This is even more clearly visible in an occlusion-based attribution map (Li *et al.*, 2016; see Supplementary Fig. S7), which shows the impact of sequentially exchanging amino acids by the unknown amino acid X and the corresponding change in the non-enzyme class prediction score. In any case, correlating known sequence properties from UniProt with interpretability methods represents a very promising direction for future work to gain deeper insights on the one hand into protein classification models and on the other hand into protein substructures.

## 5 Summary and outlook

In this work, we investigated the prospects of self-supervised pre-training for protein classification tasks leveraging the recent advances in NLP in this direction. Protein classification represents an ideal test bed for NLP methods. Most importantly, a single, universal model architecture with no task-specific modifications apart from a fine-tuning step that operates on the sequence of amino acids alone is able to reach or even exceed state-of-the-art performance on a number of protein classification tasks. This is achieved by powerful, implicitly learned representations from self-supervised pre-training, whereas most state-of-the-art algorithms make use of PSSM features obtained from BLAST database searches that scale unfavorably with dataset size. In addition, the proposed method shows particular advantages for small datasets. Differently from typical NLP tasks, the dataset creation and the evaluation procedure has to be carried out with particular care, as small differences in the procedure can have large impact on the difficulty of the classification problem and hence on the comparability of different approaches. This applies in particular to a well-defined way of handling the similarity threshold, i.e. dealing with homologous sequences that differ only by a few amino acids when splitting into train and test sets. These factors urge for the creation of appropriate benchmark datasets that convert raw data from an exemplary subset of the many existing protein classification tasks into benchmark datasets in a transparent manner that allow for a rigorous testing of ML algorithms in this setting.

Given the insights gained from the three classification tasks, we can draw the following general conclusions for generic protein classification tasks:

1. Considering the fact that *UDSMProt* was able to reach or surpass state-of-the-art performance suggests that problem-specific architectures are less important than the training procedure, at least for models that are powerful enough. This allows to design task-independent, universal classification algorithms that can be applied without much manual intervention to unseen classification tasks.
2. Redundant sequences are a valuable source of information also for downstream classification tasks. This fact is in tension with the standard practice in bioinformatics, where in many cases only representatives without the corresponding cluster assignments are presented. To ensure comparability, benchmarking datasets should always include full information to reproduce the cluster assignments used during dataset creation, which would allow at least retrospectively to reconstruct the complete dataset from a given set of representatives.
3. Bidirectional context is important, which is reflected by the fact that in all cases forward-backward-ensembles reached the best performance and in most cases improved the performance of unidirectional models considerably. Ensembling forward and backward models is in fact the simplest—although at the same time a quite inefficient—way of capturing bidirectional context. From our perspective, this represents an opportunity for approaches such as *BERT* (Devlin *et al.*, 2019; Liu *et al.*, 2019) or *XLNet* (Yang *et al.*, 2019), which are able to capture the bidirectional context directly. This might be particularly important for more complicated protein classification tasks such as sequence annotation tasks like secondary structure or phosphorylation site prediction that go beyond the prediction of a single global label.

Leveraging large amounts of unlabeled data in the form of large, in parts very well-curated protein databases by the use of modern NLP methods, represents a new paradigm in the domain of proteomics. It will be interesting to see how this process continues with the rapidly evolving algorithmic advances in the field of NLP. Apart from the huge prospects in terms of quantitative prediction performance, the recent advances in the field of explainable ML research open exciting new avenues for deeper insights into the inner structure of proteins themselves. 

## Supplementary Material

btaa003_Supplementary_Data
