# Supplementary material for: UDSMProt: universal deep sequence models for protein classification
Source: Bioinformatics. 2020 Jan 8;36(8):2401–9. doi: 10.1093/bioinformatics/btaa003 (PMC7178389; doi:10.1093/bioinformatics/btaa003)
Supplement: btaa003_Supplementary_Data [file btaa003_supplementary_data.pdf]

Supplementary Material (Online only)

1 Custom Enzyme Class Datasets EC40 and EC50

As discussed in the main text, in addition to the original *DEEPre* and *ECPred* datasets, we recreated datasets for the similarity threshold 40% and 50% by combining best practices from both [Li et al., 2018] and [Dalkiran et al., 2018]. The corresponding dataset with similarity threshold 40% relying on clusters from a prior CD-HIT [Fu et al., 2012] run is termed EC40. Compared to *DEEPre*, we slightly adapted their dataset generation procedure by clustering Swiss-Prot all at once with CD-HIT, instead of clustering enzymes and non-enzymes separately, as this mimics the way UniRef50 clusters are constructed, albeit for a similarity threshold 40% in this case. The dataset using a similarity threshold of 50% by using UniRef50-clusters is termed EC50. In both cases, we do not balance the number of enzymes and non-enzymes and restrict ourselves to proteins with a single EC-label.

Table 1 summarizes all datasets used for our experiments, namely the original data from [Li et al., 2018] and [Dalkiran et al., 2018] in the first two columns, and our replica in the last two columns.

Table 1. EC Prediction Datasets Overview

EC class	DEEPre	ECPred	EC40	EC50
Oxidoreductase	3341	8242	3781	8244
Transferase	8517	20133	9137	20139
Hydrolase	5917	16018	7987	16020
Lyase	1532	3475	1401	3475
Isomerase	1193	2883	1160	2834
Ligase	1666	4429	2165	4429
Enzymes	22166	55180	25631	55141
Non-Enzymes	22142	25333	32387	49799
Total	44336	80513	58018	104940

The EC50 and EC40 EC classification datasets are constructed according to the following procedure:

1. Acquire Swiss-Prot (2016_08 for EC40 and 2017_03 for EC50).
2. Cluster Swiss-Prot with CD-HIT (40% similarity cutoff) for EC40 or acquire UniRef50 (2017_03) for EC50 and apply it to Swiss-Prot.
3. Remove non-enzymes which have not enough experimental evidence in order to avoid misleading false negatives (annotated evidence is greater or equal to 4).
4. Remove proteins which are annotated as fragments.
5. Remove enzymes with multiple enzymatic annotations in order to obtain a single-label classification problem.
6. Filter proteins by length to include only proteins with more than 50 and less than 5000 amino acids.
7. Split by clusters into 80% training and 10% validation and 10% test set.
8. For test and validation set, we select only representatives (from CD-HIT or UniRef50 clustering) or alternatively the first member of the cluster in the case where the representative was excluded by filtering rules. Optionally a similar reduction is applied to the training set to obtain a set of non-redundant sequences.
9. Use the predefined cluster assignments of the previous step and similarly distribute all remaining Swiss-Prot clusters onto training, validation and test dataset according to split ratios 90%, 5% and 5%, respectively, to obtain a clean train-test-split on the whole Swiss-Prot dataset consistent with chosen clusters for the downstream classification task. Again, we keep only representatives for validation and test set.

The last step is important for suitable database creation for PSI-BLAST for the experiments in Section 3.2.2 and Appendix 6. One thing can already be anticipated, as a result, for significantly more sequences from the test dataset PSI-BLAST returned empty queries resulting in less informative features for the test set.

2 AWD-LSTM Parameters

Table 2 lists the AWD-LSTM hyperparameters used for language modeling and classifier finetuning.

Table 2. AWD-LSTM Parameters

Parameter	Value
Joint parameters	
Number of hidden units	1150
Number of layers	3
Embedding dimension	400
Backpropagation through time (bptt)	70
Gradient clipping	0.25
Weight decay	1e-7
Language-model-specific parameters	
Dropout (p_o,p_h,p_i,p_e,p_w)	$0.5 \cdot (0.25,0.1,0.2,0.02,0.15)$
Classifier-specific parameters	
Dropout (p_o,p_h,p_i,p_e,p_w)	$0.5 \cdot (0.4,0.2,0.6,0.1,0.5)$
Max. length (context size)	1024
Number of hidden units (head)	50

3 Detailed Discussion of Language Model Performance

We present language model results using *AWD-LSTM* language models trained on Swiss-Prot using 90% / 5% / 5% splits based on clusters for training, validation and test set, respectively. We train using redundant sequences and evaluate on a reduced dataset with a single representative sequence for each cluster. As performance metrics, we report the perplexity (as natural exponential of the test loss) and the prediction accuracy. We tokenize on the amino acid level, the resulting vocabulary comprises the following additional tokens in addition to the 20+2 canonical amino acids: `X` (unknown), `B` (either `D` or `N`), `Z` (either `E` or `Q`), <`BOS`> (marks beginning of new protein). We stress that we do not specifically aim to optimize the language model performance, which could be easily improved using appropriate postprocessing techniques [Grave et al., 2016], since it only serves as pretraining objective in this context.

At this point a comment on the different training dataset sizes is appropriate. For random splits we disregard any cluster information and distribute samples according to the ratios 90% / 5% / 5%, which obviously results in the largest training dataset. In cases where cluster information is used the splits are performed by clusters, where we sort the clusters by the number of members and distribute them onto the three sets consecutively. Finally, we also report results for a dataset, where the train-test-split used for the language model respects those of a chosen downstream classification task, in this case level 1 EC prediction on the EC50 dataset described below. This construction avoids potential data leakage from using implicit knowledge about test and validation sets that is contained in the language

Table 3. Language model performance on Swiss-Prot 2017_03 data. The downstream classification task is level 1 EC prediction on the EC50 dataset as described below.

		LM		Downstream
Split	# Train	Perpl.	Acc.	Acc.
UniRef50	316K	11.37	0.254	0.956
UniRef50 (clean)	322K	11.75	0.244	0.954
Random(64% train)	316K	7.40	0.385	0.955
Random(fwd)	499K	**6.88**	**0.409**	0.960

Table 4. Ablation study on language model performance on Swiss-Prot 2017_03 data (random split) in analogy to Table 3. The downstream classification task is level 1 EC prediction on the EC50 dataset. The first line corresponds to the setup used throughout the main text of the manuscript.

Architecture			LM			Downstream	
nl	nh	emb_sz	Perpl.	Acc.	Runtime	Acc.	Runtime
3	1150	400	**7.199**	**0.394**	33:21	**0.956**	17:18
3	575	200	9.907	0.295	12:48	0.946	8:38
3	288	100	12.807	0.213	5:52	0.910	4:32
1	1150	400	13.956	0.185	4:45	0.877	3:54

model representations in the actual downstream classification task, see Section 3.2.2 for a detailed discussion.

The results on language modeling are compiled in Table 3. As expected, the best language model performance is reached on random dataset splits with perplexities of around 7. It is the coverage of the whole dataset in terms of included clusters that is most important for language model performance. This is apparent from the comparison of (1) the language model trained on a random split, where we artificially restricted the training set to 64% of the original training set compared to (2) the UniRef50 runs. In both cases, the size of the training set is comparable but the former (1) reaches a perplexity comparable to that of the model trained on the full dataset with random splits, whereas the latter (2) only reaches a perplexity of around 11.

Even more important than the language model performance itself is, however, in our present context its impact on downstream classification tasks. This is illustrated exemplarily for the EC level 1 classification performance on the EC50 dataset as described below. Table 3 lists the downstream performance for all language models pretrained on the respective Swiss-Prot version. Interestingly, all fine-tuned classification models show a comparable performance and it is not possible to discriminate between different language models used for pretraining. The same pattern was observed across all considered classification tasks. In particular, it shows that the data leakage from inconsistent splits between pretraining and classification task is presumably small in the context of language modeling. We also experimented with the use of byte-pair-encoding to form subword units [Sennrich et al., 2015]. The language model metrics are obviously not comparable for different vocabulary sizes. Therefor, the downstream classification performance is the only meaningful metric to compare both approaches in this case. However, we did not see any significant performance improvements compared to the baseline models with single amino acids as tokens. In addition, if one tokenizes using subword units, sequence annotation tasks such as secondary structure prediction are less straightforward to handle.

Key insights from results presented in this section are the following: First, the results demonstrate that language modeling on amino acid sequence data is indeed meaningful and represents a potentially interesting domain of application for language model methods from NLP. Prediction accuracies of 40% (random split) or 25% (UniRef50 clusters) document a solid understanding of the general structure of proteins. However, the section also highlights crucial differences compared to language model evaluation in NLP. Whereas in NLP sequence similarity between train and test sequences is barely considered, it has crucial implications in proteomics. Here, it is the dataset coverage in terms of clusters rather than the nominal size of the training set, which most crucially determines the language model performance. Second, however, the different language models show no significant differences in terms of downstream classification performance. This iterates a general insight from NLP in the sense that improved language model performance does not necessarily imply improved downstream task performance.

In Table 4 we investigate the language model performance in an ablation study varying the dependence on the number of LSTM layers and the size of the hidden units. The dependence on the size of the training set was already investigated exemplarily in Table 3. In all cases the language model was trained for 30 instead of 60 epochs in the main text, which leads to a marginally worse language model performance but has no noticeable impact on the downstream performance. The first line in Table 4 corresponds to the model used in the main text. The runtime measurements refer to training on a single NVIDIA Tesla P100 GPU and should only be used as a rough orientation as for example the batch size was not adapted for smaller models, which would have lead to further speedups. The results signify that both a reduction of the size of the hidden units as well as a reduction of the number of layers leads to a considerable decrease in both language model as well as downstream performance. However, it is worth stressing again that even in the case of the full model the required time for pretraining is still very moderate and also amenable for a smaller computational budget.

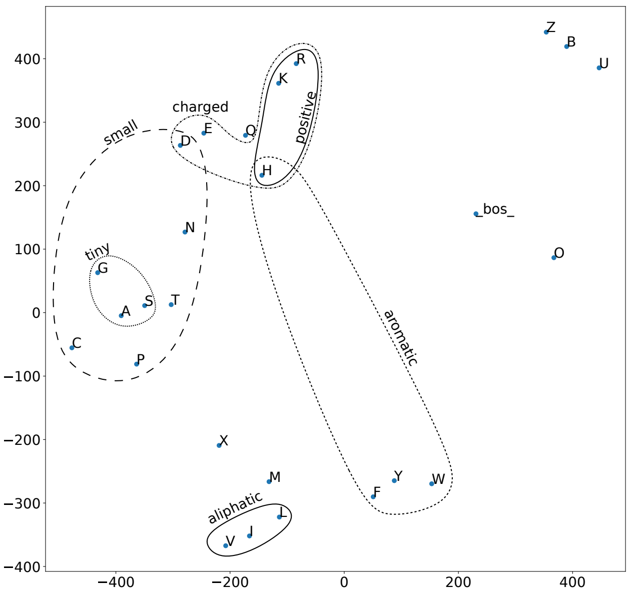

Figure 1: The t-SNE visualization of the embedding layer superimposed by clusters from [Taylor, 1986] illustrates that the amino acid embeddings learned via self-supervised pretraining align with physio-chemical properties relevant for protein properties.

4 Insights into Learned Language Model Representations

There are several levels at which one can gain further insights into the language model results from Section 3.1. As a consistency check, one can

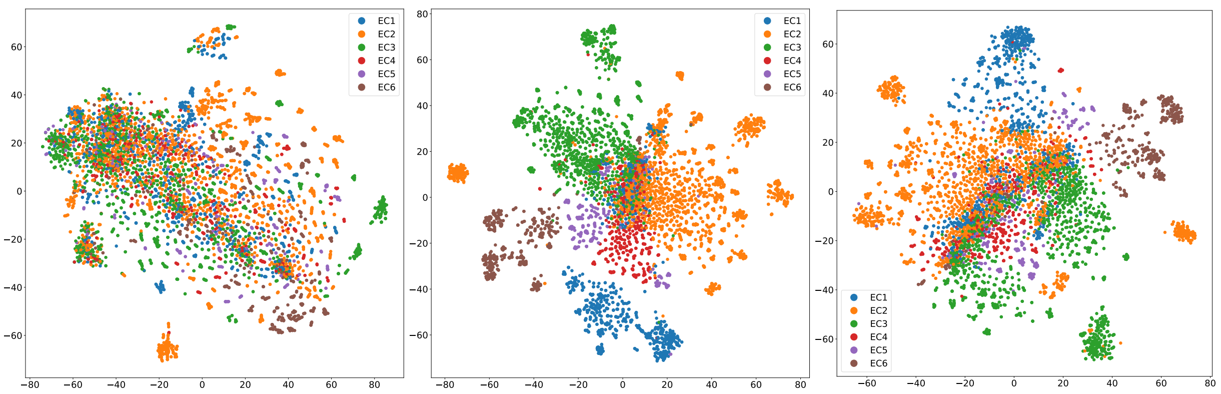

(a) After language model pretraining. (b) After language model pretraining and finetuning. (c) After training from scratch.

Figure 2: t-SNE visualization of the output of the model's encoder passed through a concat pooling layer to achieve a representation that is independent of the sequence length.

investigate the amino acid embeddings learned during self-supervised pretraining in comparison to known physio-chemical properties. Interestingly and in qualitative agreement with the findings of [Rives et al., 2019] for a transformer model, the embeddings visualized via t-SNE align very well with known groups of amino acids based on physio-chemical properties [Taylor, 1986].

This is a non-trivial and reassuring observation but leverages only a small fraction of the learned weights of the language model. To gain further details into the learned representations, we analyze the output of the model's language model encoder after passing it through the concat pooling layer in order to obtain a representation that is independent of the sequence length. For simplicity, we base our analysis on the EC level 1 classification task on the EC50 dataset. We compare the t-SNE projections of samples from the validation set and color them by their respective enzyme class to guide the eye. In Figure 2, we show the result after LM pretraining (Figure 2a), after finetuning on EC classification with LM pretraining and after training on EC classification from scratch. Interestingly, LM pretraining already leads to a local clustering of similar proteins according to the enzyme class without accessing the label information. The higher classification performance of the finetuned model with LM pretraining compared to a model trained from scratch is also understandable on an intuitive level based on the respective outputs visualized in Figure 2b and Figure 2c. LM pretraining seems to lead to a more efficient encoder that leads to more clearly separated clusters even after the concat pooling layer i.e. already before passing it to the actual classification head. This result is in line with class-wise F_1-scores of (0.955,0.956,0.952,0.930,0.954,0.991) for finetuning with LM pretraining compared to (0.934,0.950,0.930,0.905,0.930,0.980) for training from scratch, which suggest that the EC classes 1, 4 and 5 that show the largest relative increases in class-wise F_1-scores, profit from a more effective encoder as suggested by Figure 2b compared to Figure 2c.

5 Evaluation Procedure for Comparison to DEEPre and ECPred

For *DEEPre*, the reported accuracies derived from a 5-fold-cross-validation are straightforward to compute. For *ECPred*, however, we reproduced the evaluation scheme of the authors, which is tailored to binary one-vs-all-classifiers opposed to multi-class-classifiers. The scheme requires to evaluate binary classifiers that distinguish a particular EC class from other EC classes as well as non-enzymes. Finally, the mean F_1-score is reported across the six datasets. We adopt their evaluation procedure in order to be able to compare directly to their reported results. However, we decided to fit a seven-class categorical classifier (non-enzymes and six main enzyme classes) both on the concatenated training set for all EC classes. In our experiments, the performance of these classifiers was comparable or even better than the corresponding score obtained by training six independent classifiers on EC-class-specific training sets and the procedure is more in line with our approach. At this point we would like to stress that the evaluation procedure is tailored specifically to hierarchical classifiers and rather inconvenient to apply for multi-class classifiers.

6 Dependence of Data Leakage on BLAST Database Size

In this section, we investigate the data leakage effect discussed in Section 3.2.2 and Appendix 3 in more detail with focus on PSSM features. The aim is to illustrate that the overestimation of the model generalization performance by computing PSSM features on the whole dataset is not only a theoretical issue but has practical implication for downstream classification performance at least in the cases where the corresponding BLAST database is small.

For pretraining using language modeling, this issue did not have significant impact on the downstream performance. This finding is obviously a desirable property as it would otherwise require to pretrain on large datasets with train-test splits consistent with the downstream task, which defeats the purpose of using pretraining as a universal step unspecific to the choice of the downstream task. However, data leakage is always a potential issue in this context and deserves further research from our perspective to understand the practical implications for different classification tasks.

To highlight the importance of using an appropriate train-test-split also for the database on which PSI-BLAST is performed, we conducted the following experiment, where we compared two sets of features (as already described in Section 3.2.2) both trained with the same model and hyperparameters: (1) PSSM features based on the whole Swiss-Prot database (including test sequences) and (2) PSSM features on a database consisting only of sequences from the training clusters. While experimenting, we observed that the effect is consistent but barely measurable for large training data, but as the training data (and therefore the BLAST database) get smaller, the effect becomes more apparent. We considered four training sizes: 10%, 30%, 50% and 80% training size, while the test size stayed 20% (the rest of the sequences were neglected for this experiment. For each

experiment, we trained a model for level 1 EC prediction as described in Section 2.2 and shown in Section 3.2.2, where we used an early stopping criterion based on the accuracy on a validation set. Here, we used the EC40 dataset as described in Table 1.

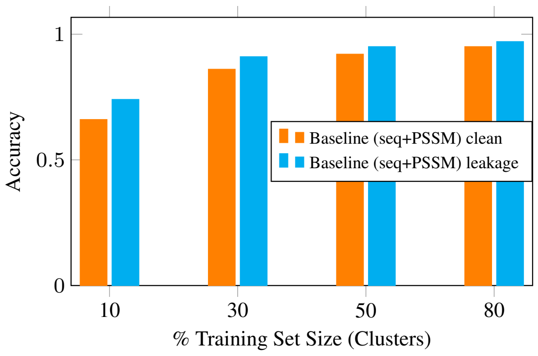

Figure 3: Dependence of EC classification accuracy on the size of the dataset used to computed PSSM features compared to a model exploiting PSSM features computed on the full dataset.

Figure 3 shows the results of this experiment. It can be seen that the effect (difference between clean and leakage) is getting more pronounced when the training dataset gets smaller. This might be a obvious fact from a machine learning point of view, but to the best of our knowledge, this has never been shown in the context of PSSM features for proteomics. From this finding, we can conclude that the results from previous literature are not overestimated strongly. Nevertheless, a fundamental difference remains when dealing with smaller datasets. In the case of small data, considering e.g. 10% of the EC40 training data, the gap in performance between a clean and a leaky procedure is 8% in accuracy, which is far from negligible. In addition, we restricted our analysis to EC classification whereas the effect might very well also depend on the considered downstream task.

7 Attribution Maps

In this section, we present the remaining attribution maps accompanying the analysis in Section 4.

```
MQLYNTLTRKKEKFIPQREGKASVYVCGITAYDLCHLGHARSSVAFDVLV
RYLRHTGLDVTFVRNFTDVDDKIIKRAGETGLTSTEVAEKYMAAFHEDMD
RLGCLRADIEPRCTQHIGEMIALCEDLISKGKAYSTASGDVYFRVRSFAS
YGKLSGRDVDDMRSGARVAPGEEKEDPLDFALWKSAKPGEPYWESPWGNG
RPGWHIECSAMSEKHLPLPLDIHGGGQDLVFPHHENEIAQTEAATGKEFA
RYWVHNGFVQVNAEKMSKSLGNFSTIRDILQGYLPETLRYFLLTKHYRSP
IDFTFDGMDEAEKNLRRIYQTLNLVENELQKTKWSAAPLPEEVLSEMDET
ERAWNEAMEDDLNTAAALGHIFGLVRLVNRIIEDKTMRKSAQARDALLRM
QSMMARWGAVLGLFTRQPAEFLREMRDCRAARRDVDTARVETLLLERQEA
RKAKDFERSDAIREELARMGVEVQDTPAGAAWDIA
```

Figure 4: Attribution map for the class EC6 for UniProt accession Q30ZH8 based on integrated gradients. The heatmap shows high relevance both on the 'HIGH' region (ITAYDLCHLGH; pos. 29-39) and the 'KMSKS' region (KMSKS; pos. 265-269).

```
MMRLRGSGMLRDLLLRSPAGVSATLRRAQPLVTLCRRPRGGGRPAAGPAA
AARLHPWWGGGGWPAEPLARGLSSSPSEILQELGKGSTHPQPGVSPPAAP
AAPGPKDGPGETDAFGNSEGKELVASGENKIKQGLLPSLEDLLFYTIAEG
QEKIPVHKFITALKSTGLRTSDPRLKECMDMLRLTLQTTSDGVMLDKDLF
KKCVQSNIVLLTQAFRRKFVIPDFMSFTSHIDELYESAKKQSGGKVADYI
PQLAKFSPDLWGVSVCTVDGQRHSTGDTKVPFCLQSCVKPLKYAIAVNDL
GTEYVHRYVGKEPSGLRFNKLFLNEDDKPHNPMVNAGAIVVTSLIKQGVN
NAEKFDYVMQFLNKMAGNEYVGFSNATFQSERESGDRNFAIGYYLKEKKC
FPEGTDMVGILDFYFQLCSIEVTCESASVMAATLANGGFCPITGERVLSP
EAVRNTLSLMHSCGMYDFSGQFAFHVGLPAKSGVAGGILLVVPNVMGMMC
WSPPLDKMGNSVKGIHFCHDLVSLCNFHNYDNLRHFAKKLDPRREGGDQR
VKSVINLLFAAYTGDVSALRRFALSAMDMEQRDYDSRTALHVAAAEGHVE
VVKFLLEACKVNPFPKDRWNNTPMDEALHFGHHDVFKILQEYQVQYTPQG
DSDNGKENQTVHKNLDGLL
```

Figure 5: Attribution map for the class EC3 for the glutaminase kidney isoform, mitochondrial with UniProt accession O94925 (isoform1) based on integrated gradients.

```
MMRLRGSGMLRDLLLRSPAGVSATLRRAQPLVTLCRRPRGGGRPAAGPAA
AARLHPWWGGGGWPAEPLARGLSSSPSEILQELGKGSTHPQPGVSPPAAP
AAPGPKDGPGETDAFGNSEGKELVASGENKIKQGLLPSLEDLLFYTIAEG
QEKIPVHKFITVSFYIFLS
```

Figure 6: Attribution map for the class NoEC for the glutaminase kidney isoform, mitochondrial with UniProt accession O94925 (isoform2) based on integrated gradients. VSFYIFLS at the end of the sequence deviates from the canonical sequence.

```
MMRLRGSGMLRDLLLRSPAGVSATLRRAQPLVTLCRRPRGGGRPAAGPAA
AARLHPWWGGGGWPAEPLARGLSSSPSEILQELGKGSTHPQPGVSPPAAP
AAPGPKDGPGETDAFGNSEGKELVASGENKIKQGLLPSLEDLLFYTIAEG
QEKIPVHKFITVSFYIFLS
```

Figure 7: Attribution map for the class NoEC for Glutaminase kidney isoform, mitochondrial with UniProt accession O94925 (isoform2) based on occlusion. VSFYIFLS at the end of the sequence deviates from the canonical sequence.
